# Unprecedented Microbial Conversion of Biliverdin into Bilirubin-10-sulfonate

**DOI:** 10.1038/s41598-019-39548-w

**Published:** 2019-02-27

**Authors:** Ryan G. Shiels, Josif Vidimce, Andrew G. Pearson, Ben Matthews, Karl-Heinz Wagner, Andrew R. Battle, Harry Sakellaris, Andrew C. Bulmer

**Affiliations:** 1 School of Medical Science, Griffith University, Gold Coast, Queensland Australia; 2 Department of Nutritional Sciences, University of Vienna, Vienna, Austria; 3 Translational Research Institute (TRI), Institute for Biomedical Innovation, School of Biomedical Sciences, Queensland University of Technology, Brisbane, Queensland 4102 Australia

## Abstract

Biliverdin (BV) possesses antioxidant and anti-inflammatory properties, with previous reports identifying protection against oxidant and inflammatory injury in animal models. Recent reports indicate that intra-duodenal administration of BV results in the formation of an uncharacterised metabolite, which is potently absorbed into the blood and excreted into the bile. This compound may be responsible for protection against inflammatory responses. This study aimed to identify novel, enterally-derived BV metabolites and determine the source of their metabolic transformation. Rat duodena and bacterial cultures of *Citrobacter youngae* were treated with BV and subsequently analysed via high performance liquid chromatography/high resolution tandem mass spectrometry to identify and characterise metabolites of BV. A highly abundant metabolite was detected in duodenal wash and bacterial culture supernatants with a 663.215 m/z (3 ppm mass accuracy) and a composition of C_33_N_4_O_9_H_36_S, which conformed to the predicted structure of bilirubin-10-sulfonate (BRS) and possessed a λ_max_ of 440 nm. Bilirubin-10-sulfonate was then synthesized for comparative LCMS/MS analysis and matched with that of the biologically formed BV metabolite. This report confirms the formation of a previously undocumented metabolite of BV in mammals, indicating that a new metabolic pathway likely exists for BV metabolism requiring enteric bacteria, *Citrobacter youngae*. These data may have important implications with regard to understanding and harnessing the therapeutic efficacy of oral BV administration.

## Introduction

Mammalian catabolism of heme by heme oxygenase-1 (HO-1) forms the linear tetrapyrrole biliverdin (BV; **1**), which is chemically reduced by biliverdin reductase (BVR) to unconjugated bilirubin (UCB). Previously, these tetrapyrroles were thought to be waste products; however, recently these compounds were found to protect from oxidant-mediated damage. Specifically, both the induction of HO-1 and the administration of **1** and related tetrapyrroles confer protection in animal models of ischemia-reperfusion injury^1,2^. Administration of **1** protects from ischemia-reperfusion injury (IRI) in the liver during transplantation in swine^3^. Furthermore, oral administration of **1** protects from Forssmann reagent induced anaphylaxis in guinea pigs^4^ and its intravenous (i.v.) administration after hemorrhagic shock and subsequent resuscitation protects against acute lung injury (ALI)^5^. Given that **1** is rapidly reduced to UCB *in vivo*^6^, it is possible that either of these molecules conferred protective effects in these models.

Intriguingly however, intraduodenal (i.d.) administration of **1** in rats leads to rapid and complete metabolism of **1** and the absorption of uncharacterised metabolites, found in the duodenum, serum, bile and urine^6^. These metabolites, which are more polar than **1** and UCB, were hypothesised to possess a rubinoid structure (λ_max_ 420–450 nm) suggesting modification or reduction at the C10 bridge of **1**. These observations suggested that oral administration of **1** instead leads to the formation of a novel active metabolite that may confer protection against anaphylaxis as reported previously^4^. The aim of this study was to identify and characterise the metabolites formed after i.d. administration of **1**^6^ and to determine whether bacteria could be responsible for its transformation. We then sought to synthesise this compound and evaluate its antioxidant activity.

## Results

**Figure 1:**
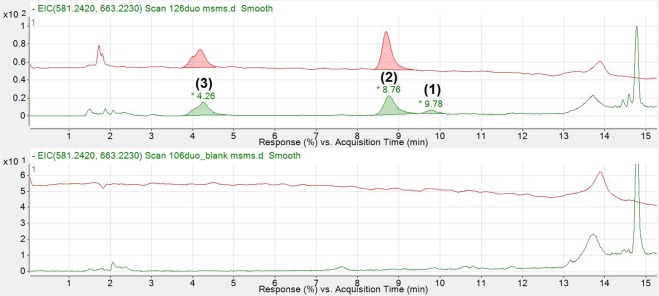
Extracted ion chromatogram of intestinal contents obtained from a rat receiving **1** (27 mg/kg), 180 mins after i.d. administration (TOP) and an animal administered saline (BOTTOM). The red chromatogram represents UV absorbance at 440 nm and serves as a point of reference for identified ions. The green chromatogram represents the extracted ion chromatogram for m/z 663.2230 ± 0.5 and 581.2420 ± 0.5. The 581.2420 m/z ion (**1**) only appears at 9.78 minutes. The compounds eluting at 4.26 min and 8.76 m min were presumed to be BV metabolites (**3** and **2** respectively).

Given that the retention time and optical characteristics of novel BV metabolites have been published previously^6^, the composition of these molecules was confirmed using tandem mass spectrometry. Figure 1 shows UV and MS chromatograms of duodenal contents taken from an anaesthetised rat 180 mins after administration of i.d. saline or **1**.

**Figure 2:**
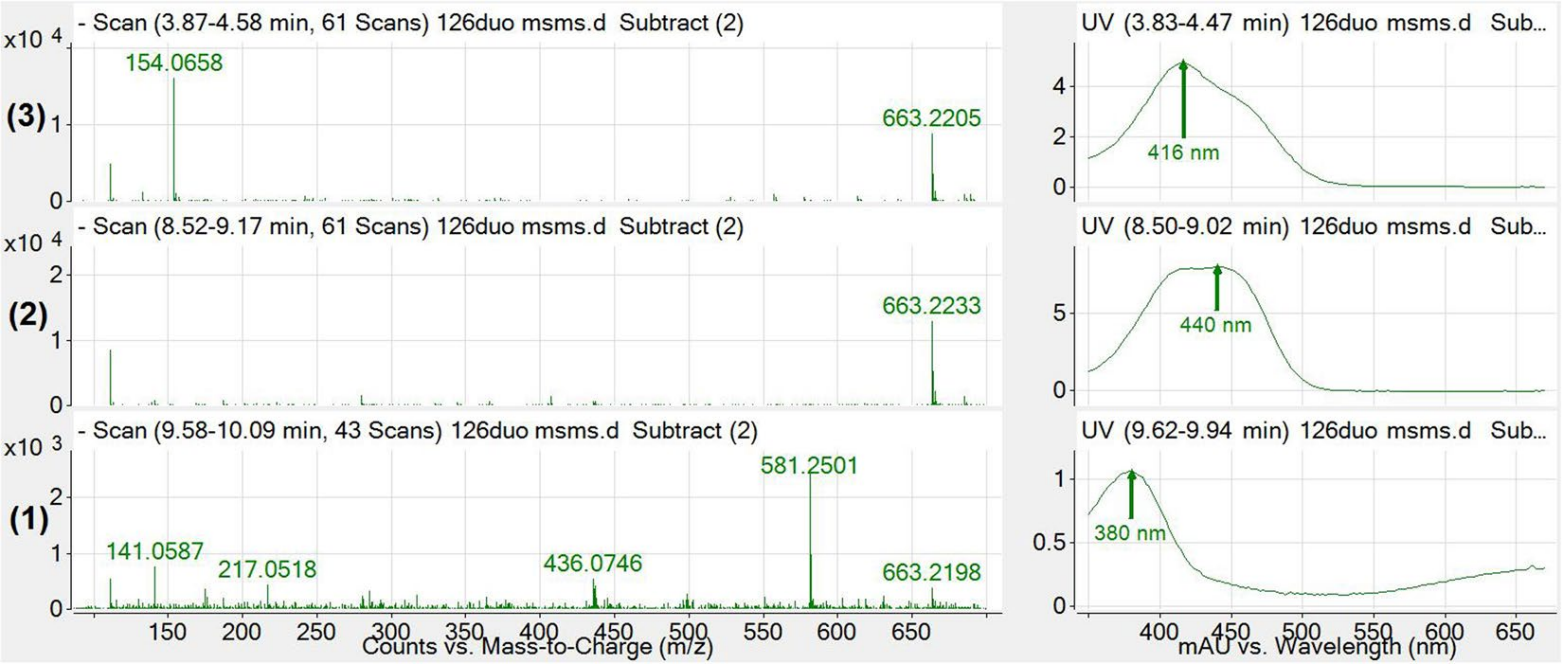
Mass spectra (left) and UV spectra (right) of each identified peak in Fig. 1. The 663.2233 and 663.2205 m/z ions were observed to be the major abundant ion under peaks **2** and **3**, with these peaks exhibiting a λ_max_ of 440 nm and 416 nm respectively. The 380 nm peak (**1**) demonstrates a mass and absorbance spectra consistent with **1**.

The three prominent peaks in **1** treated duodenal samples identified in Fig. 1 (peaks **1–3**) were then analysed for their UV and mass spectra and are displayed in Fig. 2 below.

**Figure 3:**
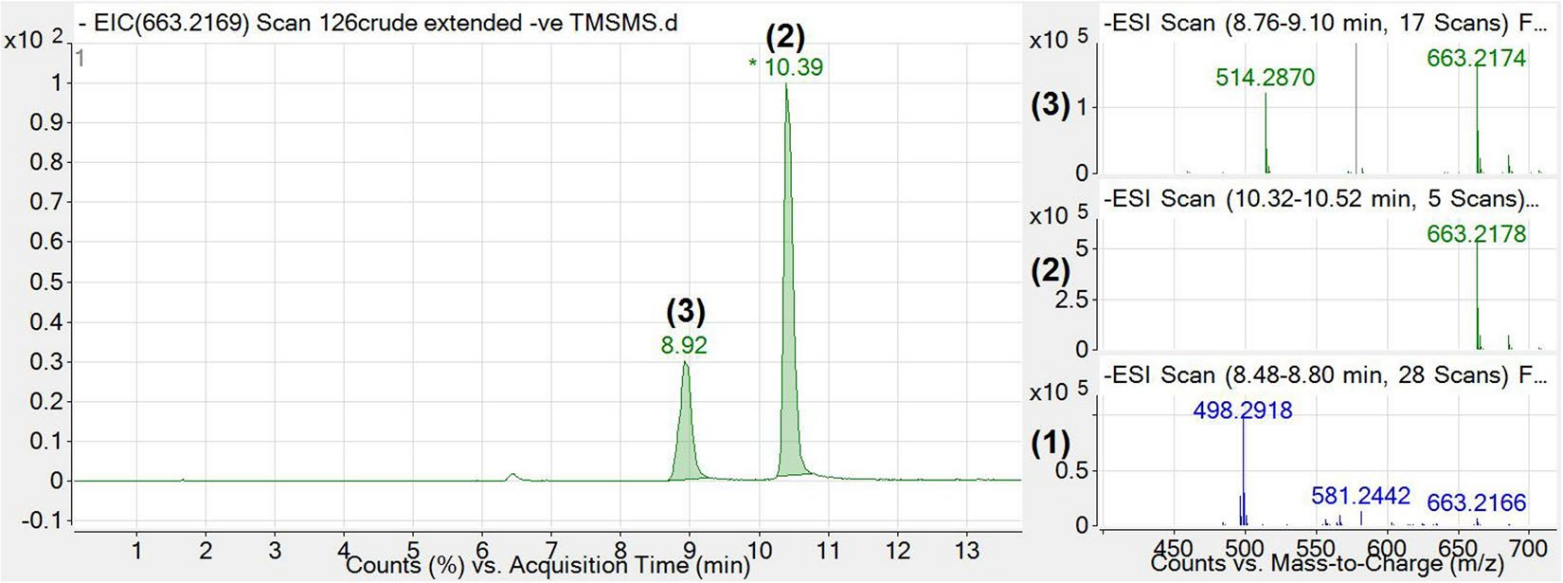
Extracted ion chromatogram (left; 663.2169 m/z ± 0.5) and MS spectra (right) of intestinal contents obtained from a rat receiving **1** (27 mg/kg), 180 mins after i.d. administration. The 663.2178 and 663.2174 m/z ions were observed to be the major abundant ion under peaks **2** and **3**, respectively (green MS spectra, top right). **1** was also identified with an m/z of 581.2442 (blue spectra, bottom right). As previously mentioned, the m/z for **1**_**calculated**_ is 581.2406 [M-H]^−^, implying a ppm mass accuracy of <7 ppm.

The m/z difference between [M-H]^−^ ions for **1**_**observed**_ (581.2501) and **1**_**calculated**_ (581.2406) was 0.0095, or 16.3 parts per million (ppm). In order to improve the mass accuracy, the PDA was removed from the flow path, the method was optimised and the instrument was cleaned and re-calibrated. Figure 3 shows MS chromatograms and mass spectra of the same BV treated duodenal sample as analysed in Figs 1 and 2, targeting the 663.2169 m/z ion for fragmentation and employing a longer, shallower gradient with a lower flow rate to optimise separation and ionisation conditions.

**Figure 4:**
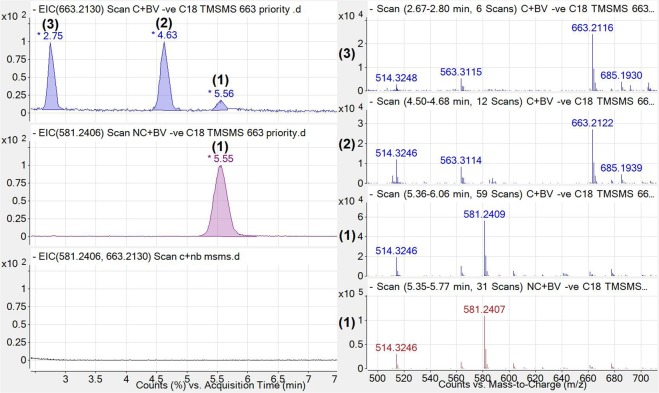
Extracted Ion Chromatogram (left) and mass spectra (right) of *C. youngae* culture treated with **1** (663.2130 m/z ± 0.5; C + BV; blue; TOP), **1** without culture (581.2406 m/z ± 0.5; NC + BV; purple; MIDDLE) and the *C. youngae* culture without **1** added (581.2406 and 663.2130 m/z ± 0.5; C + NB; black; BOTTOM). The 581.2407 and 581.2409 m/z ions were the major abundant ions under peak **1** [M-H]^−^ (<1 ppm mass accuracy), and the 663.2122 and 663.2116 m/z ions were the major abundant ion under peaks **2** and **3**, respectively.

To determine the possible source of **1** metabolism, *C. youngae*, a non-pathogenic facultative anaerobe commonly found within the gut, was cultured and treated with **1** (C + BV), without *C. youngae* culture but with **1** (NC + BV), or with *C. youngae* and nutrient broth solvent control (C + NB) for 18 hours. The supernatant was then extracted and analysed by LCMS/MS. In order to ensure adequate separation between **1** and **2**, chromatographic separation for the following bacterial culture analyses was achieved isocratically (30%A and 70%B). Representative MS chromatograms and spectra for these samples are displayed below (Fig. 4).

Having identified previously uncharacterised compounds **2** and **3**, UV, mass and fragmentation spectra (see Figs 3, 4 and 6) demonstrated that BV metabolites formed in duodenal chyme matched that of BV treated *C. youngae*. To confirm that these compounds were formed from duodenal chyme itself, 1.2 mg/mL of BV was added to fresh duodenal contents and incubated in a petri dish at 37 °C for 30 minutes. Upon addition of BV to duodenal chyme, the BV concentration decreased by ~35 μM (~22%) while BRS increased by ~40 μM, suggesting a 1:1 stoichiometric reaction (see Supplementary Figure 1). Furthermore, incubation of BV without duodenal chyme did not result in BRS production.

During collision induced dissociation (CID) of the 663.217 m/z ion (Fig. 7), a shift from 663.217 m/z [M-H]^−^ to 581.244 m/z [M-H-H_2_SO_3_]^−^ was observed, with the loss of 81.973 mass units occurring, equivalent to the neutral loss of H_2_SO_3_. This assisted in a prediction that the novel BV metabolites **2** and **3** were bilirubin-10-sulfonate (BRS). In order to confirm this prediction, a bilirubin-10-sulfonate synthesis was performed (purity > 95%, yield = 53%).

**Figure 5:**
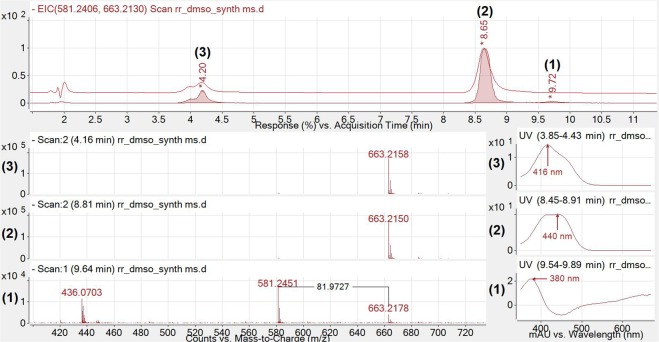
Chromatogram and related spectra of synthesized **2** (BRS; high resolution mass spectrometry mode). The red chromatogram represents UV absorbance at 440 nm and serves as a point of reference for identified ions. The superimposed chromatogram represents extracted ion chromatograms for 663.2130 and 581.2406 m/z ± 0.5. It should be noted that the 581.2451 m/z ion only appears at 9.7 minutes. The 663.2150 and 663.2158 m/z ions were observed to be the major abundant ion under peaks (**2**) and (**3**) at 440 nm and 412 nm respectively.

**Figure 6:**
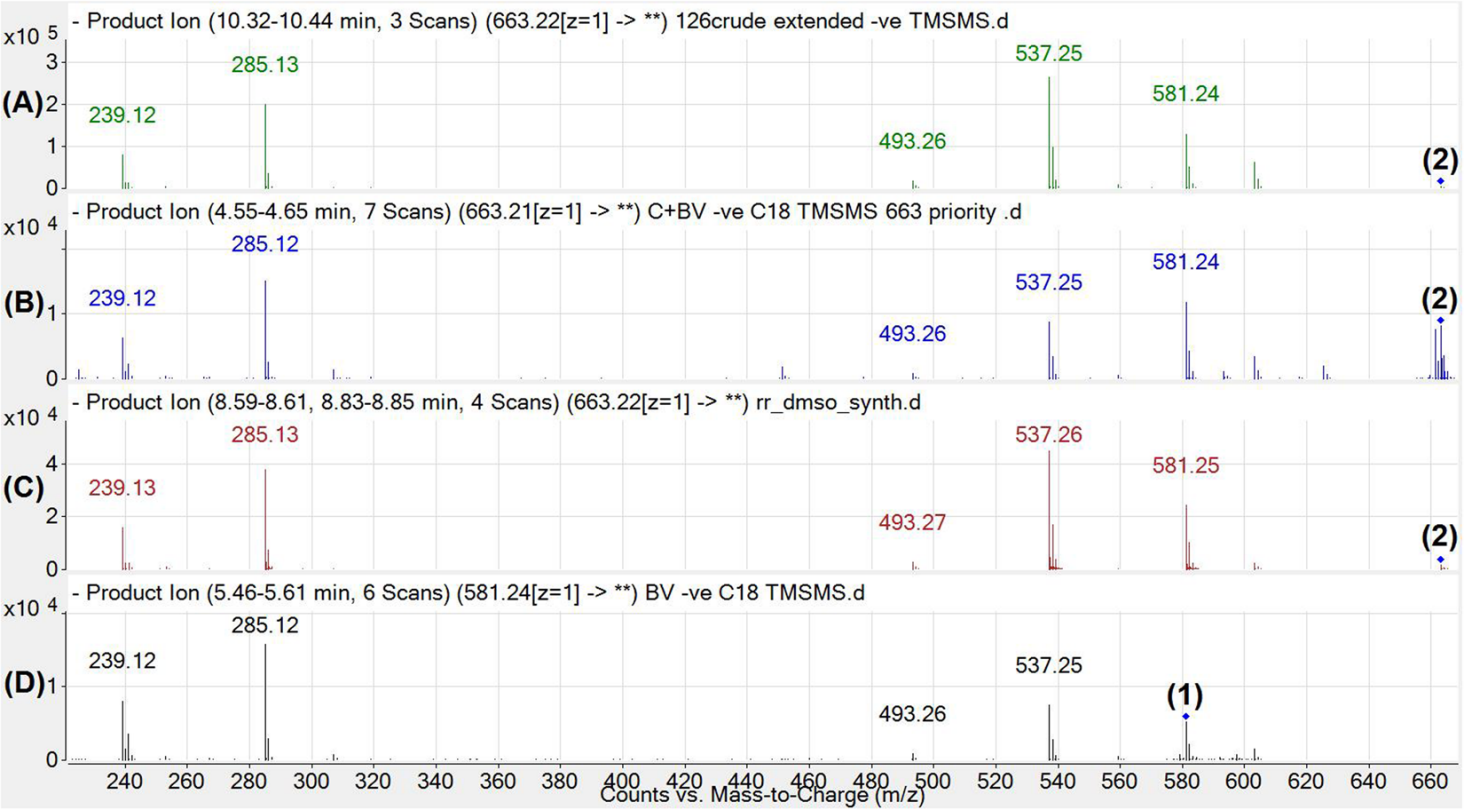
Comparison of fragmentation spectra for samples containing** 2**. The spectra show CID fragmentation at 20 V from multiple product ion scans, displayed to 2 decimal places for the targetted ~663.2150 m/z [M-H]^−^ ion collected from **1** treated rat duodena (**A**), from **1** treated bacterial assay (**B**) and from bilirubin-10-sulfonate chemical synthesis (**C**). Fragmentation of the 581.24 m/z ion (**1**) found in the bacterial assay control (no culture with **1** added) is shown (**D**) for comparison. Major fragments of 239.12/285.12/493.26/537.25/581.24 m/z were found in high abundance in all spectra. Note the m/z in this figure are displayed to 2 decimal places for clarity and ease of comparison.

Chemical synthesis and purification of **2** confirmed that the predicted metabolite of **1** had a similar mass to that of bilirubin-10-sulfonate, confirmed by using high resolution mass spectrometry (663.2150 m/z [M-H]^−^). The in source fragmentation from ESI, observed during high resolution mass spectrometry analysis of synthesised **2** (Fig. 5; bottom), suggests that a neutral loss of 81.9727 mass units occurred from the parent 663.2178 m/z ion (Fig. 5; bottom), equal to the mass of H_2_SO_3_ (<1 ppm mass accuracy). Synthesised **2** was also analysed by NMR, confirming expected shifts in ^1^H and ^13^C assignments (see Supplementary Data). LCMS analysis of synthesised **2** is shown in Figs 5 and 6 includes fragmentation analysis displayed with spectra from a duodenal sample and a *C. youngae* culture sample both treated with **1**.

As displayed in Fig. 5, the 380 nm peak (**1**) demonstrates a 581.2426 m/z ion corresponding to BV (**1**) while peaks with a λ_max_ of 440 nm and 412 nm show abundant 663.2150 and 663.2158 m/z ions corresponding to that of BRS (**2** and **3**). The bottom chromatogram shows the 663.2178 m/z ion in low abundance; note the m/z difference between **1** and **2** equalled 81.9727 corresponding to the mass of H_2_SO_3_ (calculated monoisotopic mass of 81.9724; <4 ppm mass accuracy). Some peak tailing occurred within this analysis, explaining the presence of both 581.2451 and 663.2150 m/z ions within the 9.55–9.91 **1** peak in Fig. 5.

In order to confirm the identity of BV metabolites in duodenal and bacterial samples, CID of **2** was performed (Fig. 6).

The data presented above confirmed that **2** in all samples were identical, a graphical representation of the proposed fragmentation scheme of **2** was proposed.

To determine whether **2** had reductive potential, we tested its ability to reduce ferric-tripyridyltriazine (Fe^3+^-TPTZ) to the ferrous form (Fe^2+^-TPTZ)^7^ alongside ascorbate (ASC), biliverdin (BV; **1**) and bilirubin ditaurate (BRDT) in the ferric reducing capacity of plasma (FRAP) assay (see Fig. 8).

As displayed in Fig. 8, **2** reduces 3.819 and 3.069 molar equivalents of Fe^2+^-TPTZ in aqueous and serum preparations respectively. For reference, ascorbate has been previously reported to reduce **2** molar equivalents and in this assay and reduced 1.963 and 1.991 equivalents (aqueous and plasma media) here, indicating that the assay functioned as intended.

## Discussion

**Figure 7:**
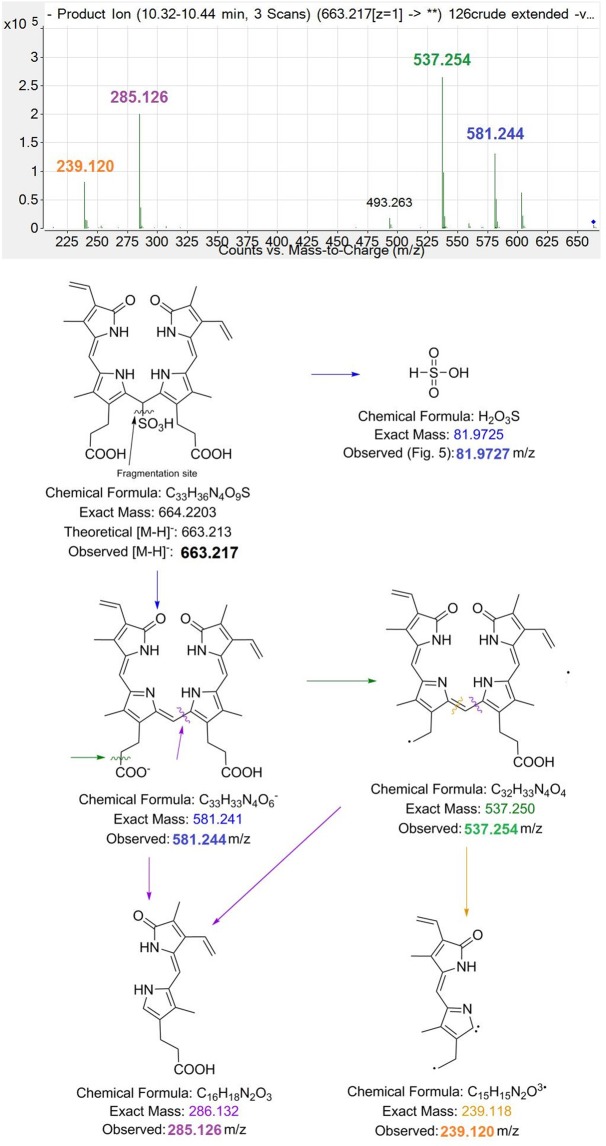
Putative structure for sulfonated BV (bilirubin-10-sulfonate; 2) with CID fragmentation pathway. The 663.215 m/z ion [M-H]^−^ first loses H_2_SO_3_. As a result of this neutral loss, the product ion 581.252 m/z [M-H-H_2_SO_3_]^−^ implies the formation of **1**. The product ions then follow a similar fragmentation pattern as **1**. Note the m/z in this figure are displayed to 3 decimal places, to demonstrate accurate mass of the fragments.

We show for the first time a new metabolic pathway for BV (**1**) in mammals, which is likely related to bacterial metabolism of **1** within the gut. Metabolism of **1** to **2** was observed *in vivo* and was confirmed in *in vitro* bacterial cultures of *C. youngae*. Formation of **2** was confirmed using HPLC, showing expected shifts in retention times and change in λ_max_ from 385 to 420–450 nm due to reduction of the C10 bridge of **1**. Furthermore, high resolution liquid chromatography-mass spectrometry determined with high confidence the product of **2**, with fragmentation spectra demonstrating removal of sulfonate and the consequent formation of **1**, which was fragmented into its dipyrrolic halves (Fig. 7).

**Figure 8:**
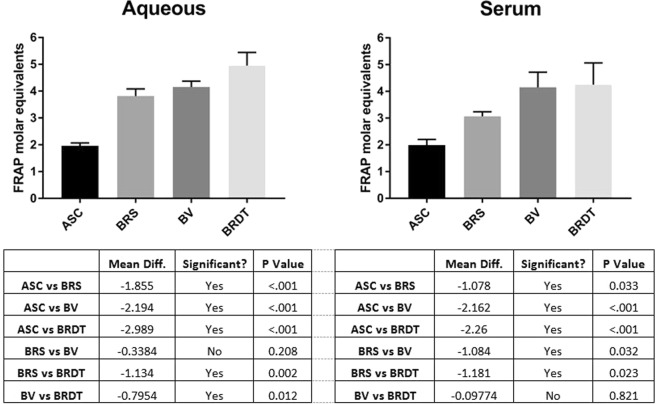
FRAP analysis of **2** (BRS), ASC, **1** (BV) and BRDT (0–100 µM; n = 3) in both aqueous (left) and serum (right). Aqueous **2** reduces 3.819 molar equivalents of Fe^3+^-TPTZ, while ASC reduces 1.963 molar equivalents. In serum, **2** only reduces 3.069 molar equivalents while there is little difference between aqueous ASC and serum ASC (1.963 vs 1.991 molar equivalents, respectively). Related tetrapyrroles (**1** and BRDT) both had more reductive potential than **2** (p < 0.05) in serum, however there was no significant difference in the reductive potential between aqueous **1** and **2**. One way ANOVA and Fisher’s LSD was used to make multiple comparisons, p < 0.05 was considered significant.

Previous reports demonstrate the formation of a BV metabolite upon i.d. administration of **1**, with chromatographic evidence suggesting the compound formed was more polar than **1** and possessed a reduced C10 bridge (λ_max_ 420–450 nm). This report sought to identify the product formed by performing additional chromatographic investigations and high-resolution tandem LCMS analysis in two independent models (*in vivo* and bacterial culture experiments). LCMS analysis revealed that two novel compounds (**2**, **3**) with differing retention times, similar UV spectra, m/z 663.215 and a similar fragmentation spectra were observed in the **1** treated rat duodenum and the **1** treated *C. youngae* culture. These data suggest that intraduodenal administration of **1** is associated with the formation of **2** and that **2** is also formed when **1** is exposed to the facultative anaerobe *C. youngae*.

HPLC and LCMS/MS confirmed the identity of **2** as bilirubin-10-sulfonate^8^ in both bacterial and duodenal samples, and NMR analysis (see Supplementary Data) verified the physico-chemical nature of chemically synthesised BRS, which shared an identical mass and CID spectra to **2** and **3**. We expect that **2** is the major biological metabolite of **1**, with the formation of **3** occurring over time when **2** is solubilised in aqueous or methanolic solution, under normal lighting conditions at room temperature.

Our previously published^6^ HPLC method used to detect BV metabolites required an *n*-dioctylamine containing mobile phase, which is incompatible with LCMS^6^. Thus, a new method was developed in order to analyse these compounds via mass spectrometry. We then observed that during both ionisation and fragmentation (Figs 5 and 6 respectively), the sulfonate group (H_2_SO_3_) at the C10 bridge of **2** was cleaved, followed by the detection of a BV 581.242 m/z ion (**1**). The neutral loss of H_2_SO_3_ has been reported previously (i.e. intestinal sulfonation of gambogic acid in the rat) and the subsequent neutral loss of H_2_SO_3_ in negative ion mode for both ESI and CID of the sulfonated compound during MS/MS analysis^9^. The 581.242 m/z ion observed in both **1** [M-H]^−^ and **2** [M-H-H_2_SO_3_]^−^ both follow similar CID for BV as previously described in the literature^10^. Interestingly, the CID spectra for **1** and **2** also feature a 285.126 m/z ion as a prominent fragment, and this fragment has been reported in the literature as a prominent fragment for UCB under similar CID conditions^11^ indicating that all 3 compounds have this dipyrrole moiety in common (Fig. 7). Chemically prepared and biologically formed **2** both exhibit similar fragmentation patterns (Fig. 6), and therefore it is possible that **1** may be metabolised into **2** in the duodenum and transported to the blood where it could contribute to reductive potential and potentially be oxidised to become **1**^12^. We confirmed that **2** contributes to reductive capacity of serum, reducing 3 molar equivalents of (Fe^3+^-TPTZ) in the FRAP assay. Therefore, future investigation into the pharmacokinetics of **2** is warranted, in addition to studying the efficacy of its administration in disease models that induce oxidative damage.

The broad specificity of anaerobic bacterial enzymes found in the human gastrointestinal tract for bile pigments has been previously demonstrated^13^ and it has been suggested that bilirubin reducing enzymes serve for the disposal of electrons produced by fermentolytic processes performed by these bacteria, however, the mechanisms of biliverdin/bilirubin metabolism are poorly understood.

*Citrobacter youngae* is widely distributed in water, soil, food, and the intestinal tract of humans and animals. It stands among several genera of the family *Enterobacteriaceae* found in mammals and respires tetrathionate (S_4_O_6_^2−^), which is formed in the intestinal mucosa of vertebrates as a result of the oxidation of thiosulfate (S_2_O_3_^2−^; an endogenous luminal sulfur compound) by reactive oxygen species (ROS) during gut inflammation^14^. The reduction of tetrathionate is catalyzed by a membrane bound electron transport chain (containing tetrathionate reductase)^15^. This process results in the reduction of tetrathionate into two molecules of thiosulfate which is in turn reduced further to sulfite (SO_3_^2−^) and hydrogen sulfide (H_2_S) via disproportionation which can provide energy for growth^16^. It is quite interesting to note that octaheme tetrathionate reductase in *Shewanella oneidensis* contains eight covalently attached heme groups^17^, which highlights the complicated interplay between mammalian heme metabolism, the use of O_2_ in mitochondria as a final electron acceptor, and the bacterial respiration of sulfates and inflammatory processes, all of which involve the structure of cyclical and linear tetrapyrroles. Based upon our limited knowledge of tetrapyrrole bacterial metabolism, we hypothesised that bacteria might sulfonate biliverdin (**1**) via the generation of bisulfite ions which are added to biliverdin at the C10 bridge, forming bilirubin sulfonate (**2**).

It is possible that *C. youngae* either enzymatically, or through the process of sulfite production occurring at the membrane during tetrathionate reduction and subsequent disproportionation into sulfites and sulfides, could catalyse the formation of **2** from **1**. We note the **2** synthesis method adopted here involved addition of sodium hydrogen sulfite to **1**^18^, lending credibility to the idea that sulfite production from bacteria may occur via a similar mechanism and result in the formation of **2**.

While there are no reports identifying the presence of endogenous **2** production in mammals, **2** has been identified in the bullfrog. McDonagh^8^ reported that the larval American bullfrog *Rana catesbeiana* does not produce UCB or bilirubin glucuronides, but rather forms a related polar linear tetrapyrrole, BRS (**2**) from BV (**1**). McDonagh also reported that **2** is excreted intact in the bile of the frog, but not in the rat^8^. This may occur because frogs lack hepatic expression of BVR^8^, meaning that **1** was available for hepatic metabolism (to **2**). Further, i.v. administration of **1** or frog hemoglobin to bullfrogs leads to biliary excretion of **2**, suggesting the hepatic formation of **2** occurs by the enzymatic addition of HSO_3_^−^ anion to **1** in the frog liver^8^. Interestingly, the bullfrog exhibits great tolerance against freezing^19^, and bullfrog tadpoles (*Lithobates catesbeiana)* exhibit high tolerance towards Paraquat toxicity, which catalyses the formation of ROS, specifically superoxide anions^20^. Given the antioxidant capacity of related tetrapyrroles^21^, **2** may form part of the antioxidant defence network in the bullfrog. We confirmed the reductive potential of **2**, demonstrating that one molecule of **2** scavenges 3.819 equivalents of FRAP oxidant (aqueous), similar to that of BRDT, supporting a conclusion that **2** may support antioxidant defence mechanisms *in vivo*.

This study suggests that oral administration of **1**, which reportedly induces protection against Forssmann anaphylaxis in Guinea pigs^4^, may instead result in **2** formation (as demonstrated in Wistar rats)^6^. **2** is rapidly absorbed and accumulates within the blood after the administration of **1**^6^ and, therefore, might represent an active metabolite of **1** and potentially protect against hypersensitivity reactions. We demonstrate that **2**, **1** and related tetrapyroles reduce Fe^3+^-TPTZ radical species and therefore may serve as a protective mechanism against inflammatory/anaphylactic mechanisms as previously reported^4,22,23^. Therefore, **2** should be tested in models of inflammation, with **1**, to discriminate relative efficacies. If **2** is determined to be efficacious, the pharmacokinetics of various **2** formulations could be tested in addition to titrating the necessary dose to maximise the efficacy of treatment.

This manuscript is the first to report gastroenteric BV (**1**) metabolism, and provides details as to the nature of this metabolite, the source of its formation and its reductive potential. These results carry importance because they may assist in explaining the anti-inflammatory effects of **1** in addition to assisting in rational drug design of anti-inflammatory drugs.

## Methods

### Reagents and Materials

Biliverdin hydrochloride was obtained from Frontier Scientific (Utah, USA). All other reagents were obtained from Sigma-Aldrich, unless otherwise stated. BV standards were stored at −30 °C (in DMSO) and biological samples were stored at −80 °C. Prior to injection, 40 µL of either standard or sample was added to 120 µL of mobile phase (50:50 20 mM ammonium acetate in 100% HPLC grade methanol: 20 mM ammonium acetate in HPLC grade H_2_O), except for duodenal samples where 120 µL of pure methanol (duodenum) was used instead of mobile phase in the same ratios.

### Synthesis of bilirubin-10-sulfonate (BRS; 2)

In a dark room and in vessels covered in foil, biliverdin.HCl (10 mg) was dissolved in 10 mL of ethanol. With the mixture, 1 mL of NaHSO_3_ solution (0.4 g; 1.92 mmol) was added and this mixture was vortexed for 30 seconds at time points 0, 15, 30 and 45 minutes. After the 45th minute, the mixture was centrifuged for 5 minutes at 4500 rpm, and the supernatant was added to 10 mL of saturated Na_2_SO_4_ with 5 µL of glacial acetic acid then added. The mixture was then vortexed for 30 seconds, and centrifuged again for 5 minutes at 4500 rpm. The supernatant was then diluted at a ratio of 1:2 (supernatant:dH2O). Under light vacuum, the mixture was passed through a C18 hypersep SPE cartridge (Thermo Fisher Scientific, Australia; 300 mg packing) that had been conditioned with 15 mL of methanol, followed by 15 mL of pure dH2O. Following the addition of the mixture, 35 mL of dH2O was passed through the column. Bilirubin-10-sulfonate was eluted from the SPE cartridge using 5 mL of methanol. Pure product was obtained after removal of methanol via centrifugal evaporation at room temperature (below 30 °C). The pure product was then analysed by mass spectrometry (ESI-MS, 663.215 m/z, Fig. 5).

### High Performance Liquid Chromatography/Mass Spectrometry – a method for the simultaneous detection of 1 & 2 present in biological matrices and sample assays

Bacterial assays, intestinal samples and standards were analysed for **1** and structurally related tetrapyrroles using HPLC-ESI/MS/MS (Agilent, California, USA). Biliverdin (**1**), bilirubin-10-sulfonate (BRS; **2**), and a primary bilirubin-10-sulfonate breakdown product (**3**) were detected using a photodiode array (380 nm, 440 nm and 412 nm respectively), in a single run. HPLC-ESI/MS/MS (LCMS/MS) analysis was performed on an Agilent 1290 HPLC (with PDA) coupled in series to an Agilent 6530 Q-TOF operating in negative ion mode using Agilent Jet Stream ESI ion source. Separation was achieved using a reverse phase C18 column (GraceSmart C18 150 × 4.6 mm, 3 μm, Grace Davidson, Australia; column oven set at 45 °C) and a flow rate of 1.0 mL·min^−1^. The initial mobile phase consisted of 50% mobile phase B (20 mM ammonium acetate in 100% HPLC grade methanol) and 50% mobile phase A (20 mM ammonium acetate in 100% HPLC grade H_2_O). A linear gradient was applied: 0–3 minutes, 50% B; 3–11 min, from 50% to 65% B; 11–12 min, from 65% to 95% B; from 12–15 min, 95% B. After 15 minutes, 50% B was run for a minimum of 7 minutes to re-equilibrate the column between analyses. Reference ions at 68.995758, 119.036320, 301.998139 and 966.000725 m/z were continually introduced into the detector along with the eluent to provide accurate reference mass correction. ESI operating conditions were: drying and sheath gas (N_2_, purity > 98%), drying gas temperature 325 °C, drying gas flow 10 L/min, nebulizer gas pressure 40 psig, sheath gas temperature 325 °C, sheath gas flow 12 L/min, capillary voltage 4 kV with the MS and MS/MS acquisition range set from 70–700 m/z. The nozzle, fragmentor, skimmer and octopole RF voltages were set at 2000, 130, 65 and 750 V, respectively. Nitrogen (purity > 99.999%) was used as the collision gas for MS/MS analysis, with collision-induced dissociation (CID) voltages set at 10, 20 and 40 V. Data were collected via targeted MS/MS analysis with a scan rate of 3 spectra/sec. A 663.2150 m/z ion was identified as the ion in highest abundance correlating with the UV peaks of unknown metabolites and was targeted as the precursor ion for CID, along with the 581.2425 m/z ion which eluted with the UV peak of **1**. **1** and **2** were also detected in series (prior to MS/MS analysis) using a photodiode array (PDA; 380 nm and 440 nm, respectively) in a single run. Agilent MassHunter software was used for UV and MS data analysis.

Prior to analysis, duodenal wash or bacterial assay samples were defrosted at 22 °C and were prepared by adding 120 μL of pure methanol to 40 μL of the sample and centrifuged (21500 × *g*; 10 min). The supernatant from each sample was then passed through a 0.22 micron PFTE syringe filter (Shimadzu, Australia), and 20 μL of the filtrate was injected via the autosampler for analysis.

### Duodenal administration of 1 to rats

Rats were administered biliverdin hydrochloride as documented previously^6^. To prepare a duodenal sample, 1 mL of sterile phosphate buffered saline was washed through the duodenum via syringe and collected. It was then centrifuged (21500 × *g*; 10 min) and the supernatant was aliquoted and immediately transferred to a −80 °C freezer for later analysis.

### Bacterial metabolism of 1

To test whether *C. youngae* metabolised **1**, 100 μL of a 1 mM solution of **1** or equivalent volume of diluent (2% DMSO + 98% 100 mM TRIS-HCl 7.6 pH) was added to a *C. youngae* culture at 1: 1 ratio and was incubated at 37 °C for 60 mins. The reaction was then quenched with 800 µL of −20 °C methanol (MeOH). To ensure consistency and repeatability between experiments the culture broths were inoculated for a period of 18 hrs, the bacterial content of which was standardised to 0.28 OD by measuring optical density with a spectrophotometer (POLARstar, Germany) at 600 nm. After completion of the assays, the samples were centrifuged (30000 × *g*; 80 seconds) and the supernatant was aliquoted and immediately transferred to a −80 °C freezer for later analysis.

Appropriate control assays were prepared in the same manner, with one excluding **1** (culture with no **1**) and another not including bacterial culture including **1** (no culture with **1**).

### Duodenal chyme metabolism of 1

To confirm that the duodenal content, and potentially bacteria therein was responsible for forming **1**, an 8 cm length of duodenum, cut 2 cm distal to the pyloric sphincter, was removed from a naïve Wistar rat. To one end of the freshly excised duodenum, a 5 mL syringe was attached and 5 mL of phosphate buffered saline was washed through it with the resulting liquid collected in a petri dish. A control sample of 5 mL PBS added directly to a second petri dish prepared at the same time. A 500 μL sample was collected prior to the addition of sodium biliverdinate from both petri dishes and analysed for its BV and BRS concentration. To both dishes, 500 μL of 1.2 mg/mL sodium biliverdinate was then added and both dishes were incubated in the dark at 37 °C. At 1, 5, 15 and 30 minutes, a 500 μL sample was collected. 40 μL of each sample was immediately prepared for analysis of BRS and BV via LCMS.

### FRAP assay

The ferric reducing ability of plasma (FRAP) was determined by the method of Benzie and Strain^7^ and performed on a COBAS Integra 400 analyzer (Roche Diagnostics, Australia). Three independent dose response experiments were conducted, with each concentration of each bile pigment tested in duplicate in each experiment. The sodium salt of biliverdin was prepared as previously published^6^. All other compounds were solubilised in distilled H_2_O at stock solutions of 4 mM and were diluted further for experimental testing (final concentrations 0–100 µM). All solutions were covered in foil and lights dimmed during experimental testing. All solutions were made in either ddH2O or human plasma obtained with informed consent from a single healthy donor and repeated in triplicate, with all methods carried out in compliance with relevant guidelines and regulations. Reductive equivalents were determined by fitting a linear regression to the dose response relationship with the slope of that relationship quantifying the reducing equivalents per mole of bile pigment.

### NMR analysis

^1^H and ^13^C NMR spectra were recorded at 298 K on an Avance 300 MHz spectrometer (300 and 75 MHz, respectively; Bruker BioSpin). ^1^H-^1^H correlation spectroscopy (COSY) and ^1^H-^1^3C heteronuclear single quantum coherence (HSQC) were used to confirm ^1^H and ^13^C assignments. Signals are reported as chemical shift (δ in ppm) relative to (CD_3_)_2_SO (^1^H NMR: δ = 2.50 ppm; 13 C NMR: δ = 39.52 ppm). Coupling constants (*J*) are reported in Hz and can be found in the Supplementary Information.

### Animal Use

All animal care and experimental procedures complied with the Guidelines of the Australian National Health and Medical Research Council and were approved by the Ethical Committee of the University of Queensland.

### Human Tissue

Human blood was obtained with informed consent from all subjects under permission from the Griffith University Human Research Ethics Committee, all methods were carried out in accordance with relevant guidelines and regulations.

## Supplementary information

shiels_et_al Supplementary Information
