# Supplementary material for: Unprecedented Microbial Conversion of Biliverdin into Bilirubin-10-sulfonate
Source: Sci Rep. 2019 Feb 27;9:2988. doi: 10.1038/s41598-019-39548-w (PMC6393463; doi:10.1038/s41598-019-39548-w)
Supplement: Supplementary file 1 — shiels_et_al Supplementary Information [file 41598_2019_39548_MOESM1_ESM.docx]

**Unprecedented Microbial Conversion of Biliverdin into Bilirubin-10-sulfonate**

Ryan G. Shiels^1^, Josif Vidimce^1^, Andrew G. Pearson^1^, Ben Matthews^1^, Karl-Heinz Wagner^2^, Andrew R. Battle^3^, Harry Sakellaris^1^ and Andrew C. Bulmer^1*^

^1^Menzies Health Institute Queensland and School of Medical Science, Griffith University, Gold Coast, Queensland, Australia

^2^Department of Nutritional Sciences, University of Vienna, Vienna, Austria

^3^Translational Research Institute (TRI), Institute for Biomedical Innovation, School of Biomedical Sciences, Queensland University of Technology, Brisbane, Queensland, 4102, Australia.

[*a.bulmer@griffith.edu.au](mailto:*a.bulmer@griffith.edu.au)

**Supplementary information**

**Duodenal chyme metabolism of 1**

Supplementary Figure 1: Formation of BRS (2) over time, after addition of BV (1) to duodenal chyme.

**Sodium bilirubin-10-sulfonate (2)**







^1^H NMR (400 MHz, DMSO-*d*_6_): δ10.21 – 10.08 (m, 2 H, 2 × COOH); 6.81 – 6.72 (m, 1H, H-18α); 6.52 (dd, 1H, *J*_3α,3β_ *_cis_* 17.5, *J*_3α,3β_ *_trans_* 11.5 Hz, H-3α); 6.14 (dd, 1H, *J*_3β_ *_cis,_*_3β_ *_trans_* 2.7 Hz, H-3β *cis*); 6.07 (br s, 2 H, H-5, H-15); 5.61 – 5.57 (m, 2 H, H-18β); 5.24 (dd, 1 H, H-3β *trans*); 5.19 (s, 1H, H-10); 4.13 – 2.90 (br s, 4 H, 4 × NH); 2.70 – 2.45 (m, 6 H, H8α, H-8β, H12α); 2.20 – 1.93 (m, 11 H, H-7α, H-12β, H-13α, H-17α); 1.85 (s, 3 H, H-2α). ^13^C NMR (100 MHz, DMSO-*d*_6_): δ178.1, 172.0, 171.2, 171.2 (C-1, C-8 γ, C-12γ, C-19); 142.7, 140.9, 130.8, 130.0, 129.8, 129.6, 127.8, 127.5, 124.6, 123.6, 123.5, 123.4, 123.3, 122.7, 122.7, 122.2, 121.7, 121.5, 117.9 (C-2, C-3, C-3α, C-3β, C-4, C-5, C-6, C-7, C-8, C-9, C-11, C-12, C-13, C-14, C-15, C-16, C-17, C-18, C-18α, C-18β); 101.0, 100.3 (C-5, C-15); 54.8 (C-10); 36.1 (C-8β, C-12β); 21.6 (C-2α); 20.4 (C-8α); 20.4 (C-12α); 10.0, 10.0, 9.8 (C-7α, C-13α, C-17α).

HRMS calculated for C_33_H_35_N_4_NaO_9_S [*M*-Na]^‑^, 663.212477; found ESIMS (*m*/*z*): 663.2150 [*M*-Na]^‑^. (<5 ppm mass accuracy)
